# Human Coronaviruses HCoV-NL63 and HCoV-HKU1 in Hospitalized Children with Acute Respiratory Infections in Beijing, China

**DOI:** 10.1155/2011/129134

**Published:** 2011-07-21

**Authors:** Li-Jin Cui, Chen Zhang, Ting Zhang, Rou-Jian Lu, Zheng-De Xie, Ling-Lin Zhang, Chuan-Yan Liu, Wei-Min Zhou, Li Ruan, Xue-Jun Ma, Wen-Jie Tan

**Affiliations:** ^1^National Key Laboratory of Molecular Virology and Genetic Engineering, National Institute for Viral Disease Control and Prevention, China CDC, Beijing 102206, China; ^2^Virology Laboratory, Beijing Children's Hospital, Beijing 100045, China

## Abstract

The human coronaviruses (HCoVs) HCoV-NL63 and HCoV-HKU1 are two recently discovered coronaviruses that circulate widely and are associated with acute respiratory infections (ARI). We detected HCoV-NL63 and HCoV-HKU1 in specimens collected from May 2008 to March 2010 from patients with ARI aged <7.75 years of age attending the Beijing Children's Hospital. Thirty-two (8.4%) and 57 (14.9%) of 382 specimens tested positive for HCoV-NL63 and HCoV-HKU1, respectively, by real-time RT-PCR. Use of a Luminex xTAG RVP Fast kit showed that coinfection with respiratory syncytial virus and parainfluenza 3 virus was common among patients infected with either virus type. In HCoV-HKU1-infected patients, the predominant clinical symptoms were cough, fever, and expectoration. In HCoV-NL63-infected patients they were cough, fever, and rhinorrhea. Phylogenetic studies showed that the HCoV-HKU1 nucleoprotein gene was relatively conserved compared to NCBI reference sequences, while the 1ab gene of HCoV-NL63 showed more variation.

## 1. Introduction

Before the outbreak of severe acute respiratory syndrome caused by the SARS coronavirus in 2003, human coronaviruses (HCoVs) had not been considered harmful respiratory pathogens. The outbreak of SARS renewed interest in this virus family and resulted in the identification of two additional HCoVs, HCoV-NL63 and HCoV-HKU1. HCoV-NL63, a member of the group I coronaviruses, was first detected in a child with bronchiolitis in The Netherlands in 2004 [1]. HCoV-HKU1, a group II virus, was detected in an adult with chronic pulmonary disease in Hong Kong in 2005 [2]. It is now recognized that both these viruses have a worldwide circulation and are associated with human respiratory tract infections. In China, several groups have demonstrated the existence of HCoV-NL63 and HCoV-HKU1 as potential respiratory pathogens of infants and the elderly [3–5]. However, more precise data regarding their epidemiology, and genetic characteristics is lacking in mainland China. In this study, we screened for the presence of HCoV-NL63 and HCoV-HKU1 in children with acute respiratory infection admitted to the Beijing Children's Hospital in an effort to gain a better understanding of the seasonality, epidemiology and genetic diversity of these viruses in a city with a population of more than 22 million.

## 2. Materials and Methods

### 2.1. Clinical Specimens

A total of 382 nasopharyngeal aspirates (NPAs) were collected from hospitalised children with a diagnosis of pneumonitis or bronchopneumonia and a body temperature above 38°C. The children were admitted to the Beijing Children's Hospital between May 2008 and March 2010 (a 23-month period). The parents of all children involved in the study provided informed consent for specimen collection and testing. The children had a median age of 1.1 years (range 1 month to 7.75 years). NPAs were collected into virus transport medium and stored at −70°C before being tested.

### 2.2. RNA Extraction and cDNA Synthesis

Nucleic acid was extracted using QIAamp MinElute Virus Spin kits (Qiagen, Mississauga, Ontario, Canada) according to the manufacturer's instructions. cDNA was synthesized from 10 *μ*L of RNA eluate using random hexamer primers (TaKaRa Dalian, China) and SuperScript II Reverse Transcriptase (Invitrogen Carlsbad, Calif, USA).

### 2.3. Real-Time RT-PCR Assays

Two real-time RT-PCR assays targeting conserved regions of the N and 1ab genes of both viruses were developed and used in parallel. The primer and probe sequences for HCoV-NL63 were as follows: 

NL63-N forward primer 5′-AGGACCTTAAATTCAGACAACGTTCT-3′; reverse primer 5′-GATTACGTTTGCGATTACCAAGACT-3′ [6]*; *TaqMan probe 5′-FAM-TAACAGTTTTAGCACCTTCCTTAGCAACCCAAACA-TAMRA-3′. NL63-1ab forward primer 5′-TGTTGTAGTAGGTGGTTGTGTAACATCT-3′; reverse primer 5′-AATTTTTGTGCACCAGTATCAAGTTT-3′; TaqMan probe 5′-FAM-CAATTGTTAGTGAGAAAATTTCTGTTATGGA-TAMRA -3′ [7]. 

The primers and probes for HCoV-HKU1 were as follows:

HKU-1-N forward primer 5′-AGTTCCCATTGCTTTCGGAGTA-3′; reverse primer 5′-CCGGCTGTGTCTATACCAATATCC-3′; TaqMan-MGB probe, 5′-FAM-CCCCTTCTGAAGCAA-MGB-3′ [8]; HKU-1-1ab forward primer 5′- CCATTACAAGCCATAAGAGAACAAAC-3′; reverse primer 5′-TATGTGTGGCGGTTGCTATTATGT-3′; TaqMan probe 5′-FAM-TTGCATCACCACTGCTAGTACCACCAGG-TAMRA-3′ [9]. 

To assess the sensitivity of the real-time RT-PCR assays, amplicons of the N and 1ab genes were cloned into pET-9a (QIAGEN) and pGEM-T Easy (Promega Corporation, USA) vectors, respectively, followed by* in vitro* transcription using the T7 RiboMAX Express Large Scale RNA Production System (Promega). Standard curves for the N and 1ab gene assays were constructed using serial log dilutions (from 10^1^ to 10^7^ copies) of the transcribed RNA fragments. The lower limit of detection of each real-time RT PCR assay was 100 copies/20 *μ*L, with intra-assay coefficients of variability (CVs) between 0.45 and 1.02% (*n* = 3) and interassay CVs between 0.68 and 2.24%.

### 2.4. Detection of HCoV-NL63 and HCoV-HKU1 in Clinical Specimens

Specimens were tested for presence of HCoV-NL63 or HCoV-HKU1 by real-time RT-PCR using a TaqMan RNA-to-CT 1-Step kit (Applied Biosystems, USA) and an ABI Prism 7000 TaqMan machine (Applied Biosystems, USA). 10^4^ copies of N gene and 1ab gene transcripts were used as positive controls in each run. The amplification conditions were 48°C for 15 min, followed by 40 cycles of 94°C for 15 sec and 60°C for 15 sec. For each virus, when the C_T_ value generated was less than 38, the specimen was considered positive. When the C_T_ value were relatively high (38 ≤ C_T_ **<** 40), the specimen was retested twice and considered positive if the C_T_ value of any retest was less than 40.

### 2.5. Detection of Common Respiratory Viruses

All HCoV-NL63- and HCoV-HKU1-positive samples were tested for other respiratory viruses using luminex xTAG RVP Fast test (Luminex Trading, Shanghai, China) on a Biorad Bio-Plex 200 (BioRad, Hercules, Calif, USA) suspension array system.

### 2.6. Phylogenetic Analysis

Fifteen sequences (251 nucleotides (nt)) of the HCoV-NL63 replicase polyprotein 1ab gene and five sequences (951 nt) of the HCoV-HKU1 nucleoprotein (N) gene were derived from positive samples using conventional PCR and sequencing and subjected to phylogenetic analysis. Genbank sequences with the highest identity (lowest expect (E) value) in blastn searching were used as references for this analysis, together with common type strain HCoV sequences. Nt sequences AY597011.2, AY56787.2 and DQ415910.1 were used as references for the HCoV-NL63 analysis. Sequences AY567487.2, AY518894.1, DQ445911.1, GU068568.1, DQ846901.1, and EF081296.1 were applied in the HCoV-HKU1 analysis. AF304460, representing the sequence of HCoV-229E, was also included. Multiple sequence alignment was performed with clustalW. Phylogenetic trees were constructed using Neighbor-Joining. Bootstrap values were calculated on 1000 replicates of the aligned data sets.

## 3. Results

### 3.1. Incidence and Clinical Symptoms Associated with HCoV-NL63 and HCoV-HKU1 Infections in Hospitalized Children

Of the 382 specimens tested, 32 (8.4%) were positive for HCoV-NL63 and 57 (14.9%) were positive for HCoV-HKU1. Testing and clinical data for patients with these infections is summarized in Table 1. Most patients infected with either HCoV-NL63 (65.6%) or HCoV-HKU1 (77.2%) were less than 1 year of age at the time of investigation. Both viruses were more frequently detected in males (Table 1). The 4 most frequent clinical symptoms for both viruses were cough, fever, rhinorrhea, and expectoration. However, expectoration was a more frequent symptom in HCoV-HKU1-infected patients (54.4 versus 25.0%). The admitting diagnosis in more than 80% of children was pneumonia and bronchopneumonia, irrespective of whether the infecting virus was HCoV-NL63 or HCoV-HKU1.

### 3.2. Epidemiology of HCoV-NL63 and HCoV-HKU1 Infections

**Figure 1:**
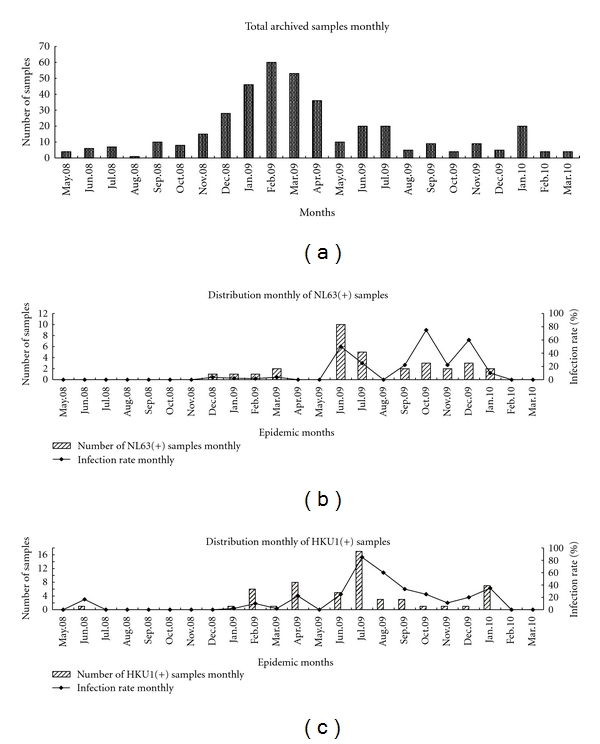
Temporal distribution of HCoV-NL63 and HCoV-HKU1 infections in hospitalized children between May 2008 and March 2010. (a) the number of specimens collected from children admitted with acute respiratory infections to the Beijing Children's Hospital; (b) the number of positive specimens and percentage positive by month for HCoV-NL63; (c) the number of positive specimens and percentage positive by month for HCoV-HKU1.

The seasonal distributions of HCoV-NL63 and HCoV-HKU1 from May 2008 to the end of March 2010 were similar, with heightened activity occurring at times when the number of samples collected from children with respiratory infections was declining (Figure 1). Peak incidences of both viruses occurred in the summer of 2009 but not in 2008. Increased HCoV-NL63 infections were also detected in the fall and winter months of 2009, in contrast to 2008 when only a small number of cases were recognized in winter. A similar distribution occurred for HCoV-HKU1.

### 3.3. Coinfection of HCoV-NL63 and HCoV-HKU1 with Other Respiratory Viruses

Testing of HCoV-positive samples using a Luminex xTAG RVP Fast system revealed that many were coinfected with other common respiratory viruses (Table 2), in particular respiratory syncytial virus (RSV) and parainfluenza virus type 3. Coinfections involving multiple HCoVs were also detected (Table 2). Only 13 of 57 (14.0%) HCoV-HKU1-positive patients and 8 of 32 (25%) HCoV-NL63-positive patients tested negative for other respiratory viruses.

### 3.4. Phylogenetic Analysis of HCoV-NL63 and HCoV-HKU1 Strains

**Figure 2:**
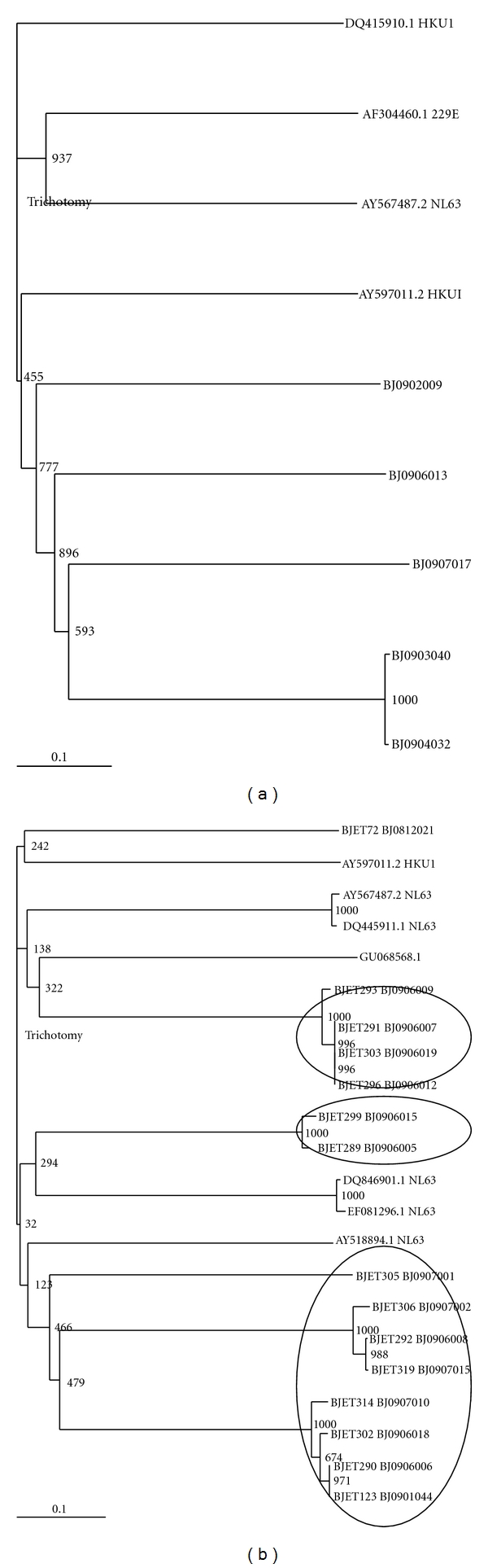
Phylogenetic analysis of the nt sequences of HCoV-HKU1 (a) and HCoV-NL63 (b). The N genes were amplified from positive samples collected between November 2008 and October 2009. Trees were constructed using Neighbor-Joining methods. Local HCoV strains are indicated by the designation BJ and are all from 2009. Bootstrap values are indicated.

The results of phylogenetic analyses are shown in Figure 2. Based on N gene analysis, the HCoV-HKU1 strains clustered together with the reference strain sequence AY597011.1 (Figure 2(a)), although the number of sequences derived from clinical material and controls used in the analysis was low. The 15 strains of HCoV-NL63 were more widely distributed on the tree when the 1ab (replicase) sequence was analysed (Figure 2(b)). Although shown to be an HCoV-NL63 strain by PCR, BJET72 was not related to any reference NL63 genes used in the analysis. The other 14 strains clustered into at least 3 distinct monophyletic groups, one related to EF081296.1 and DQ846901.1, both originally detected in the USA and China in 2007, a second close to AY518894.1, a genotype A strain found in The Netherlands, and a third close to GU068568, also detected in China in 2007. Possible evidence of subclustering within the group 2 sequences could be seen.

## 4. Discussion

Infection with HCoV-NL63 and HCoV-HKU1 has been well documented in children presenting with respiratory tract infections, although the epidemiology and clinical course of these recently identified viruses have not been fully elucidated in China. Our data indicate that HCoV-NL63 and HCoV-HKU1 are present in hospitalized children in Beijing and cause significant morbidity. Several groups have reported that these viruses can be detected in patients with acute respiratory tract infections [10], including among Chinese children and adults living in Hong Kong [3, 4]. We found that HCoV-NL63 and HCoV-HKU1 were present in 8.4% and 14.9%, respectively, of respiratory samples collected over a 23-month period. This detection rate was higher than that in previous reports from Hong Kong and other countries [11–16], probably due to the increased detection sensitivity of real-time RT-PCR technologies targeting the N and 1ab genes.

In temperate climates, most HCoV-NL63 infections seem to occur in winter and early spring, including in Canada [6, 17] and France [18]. In Hong Kong, most HCoV-NL63 infections were detected in the spring and summer of 2002, in the early summer and autumn of 2004–2005, and in the autumns of 2005-2007 [19]. In the present study, HCoV-NL63 infections peaked in the summer, fall, and winter months of 2009, but there was little activity at any time during 2008. Similarly, HCoV-HKU1-positive specimens were found frequently in winter-spring season in countries with temperate climates, including the USA and Italy [20–22]. Our data suggests that the seasonal circulation of HCoV-NL63 and HCoV-HKU1 in temperate regions is dissimilar to that occurring in the tropics. It is worthwhile noting that more specimens from patients with ARI were collected during the winter months of 2008 to spring months of 2009 than at any other time during the study, but the infection rates of both viruses were relatively low during this time.

Being RNA viruses, HCoV-NL63 and HCoV-HKU1 have a high degree of genetic diversity, although studies on the sequence variability of HCoV-NL63 isolates in Asian countries are limited. Our N gene sequencing showed that HCoV-HKU1 strains circulating in Beijing are genotype A viruses, although the numbers analysed were small. The 1ab gene of HCoV-NL63 viruses showed more diversity. However, further sequencing of other genes is needed to enable assessment of the overall genetic diversity of HCoV-NL63 in Beijing and to confirm the genotype-specific assignment for these viruses, since this depends on the gene sequence analyzed [23]. 

Studies of multiple respiratory viruses indicate that although single infections with HCoVs occur, coinfection with RSV and parainfluenza 3 viruses is common in both HCoV-HKU1- and HCoV-NL63-positive patients. This makes it difficult to clarify the role of coronaviruses in childhood pneumonia or bronchopneumonia. Future studies using real-time RT-PCR and sample collection over several consecutive years will be helpful in providing further insights into these etiologic agents of respiratory disease in China.

## Figures and Tables

**Table 1:** HCoV-NL63 and HCoV-HKU1 detection in 382 children with acute respiratory infections in Beijing, China, May 2008 to March 2010. Age, gender, and clinical details are listed for those positive for either virus.

Parameter	No. events detected in 382 children	
	tested (%)	
	NL63 (*n* = 32)	HKU1 (*n* = 57)
Age (months)		
≤12	21 (65.63)	44 (77.19)
12–35	10 (31.25)	13 (22.81)
36–93	1 (3.13)	0
Gender		
Male	21 (65.63)	43 (75.4)
Female	11 (34.37)	14 (24.6)
Clinical symptoms		
Cough	**31 (96.89)**	**56 (98.25)**
Fever	**24 (75.00)**	**36 (63.16)**
Rhinorrhea	**9 (28.13)**	18 (31.58)
Expectoration	8 (25.00)	**31 (54.39)**
Dyspnea	4 (12.50)	8 (14.04)
Nasal obstruction	2 (6.25)	9 (15.79)
Sore throat	3 (9.38)	2 (3.51)
Wheeze	0	2 (3.51)
Admitting diagnosis		
Bronchopneumonia	10 (31.25)	18 (31.58)
Pneumonia	16 (50.00)	29 (50.88)
Bronchitis	4 (12.50)	5 (8.77)
Peribronchitis	1 (3.13)	3 (5.26)
Uncertainty	1 (3.13)	2 (3.51)

**Table 2:** Coinfection of HCoV-positive samples with other respiratory viruses.

Respiratory viruses	No. coinfection of other respiratory viruses			
	HCoV-HKU1 positive, *n* (%)*n* = 57		HCoV-NL63 positive (%) *n* = 32	
HCoV-HKU1	57	(100.00)	11	(34.38)
HCoV-NL63	11	(19.30)	32	(100.00)
HCoV-OC43	2	(3.51)	2	(6.25)
HCoV-229E	1	(1.75)	0	(0.00)
Influenza A	4	(7.02)	4	(12.50)
RSV	19	(33.33)	9	(28.13)
Parainfluenza 1	4	(7.02)	2	(6.25)
Parainfluenza 2	0	(0.00)	0	(0.00)
Parainfluenza 3	15	(26.32)	6	(18.75)
Parainfluenza 4	0	(0.00)	1	(3.13)
Adenovirus	0	(0.00)	3	(9.38)
Human metapneumovirus (hMPV)	7	(12.28)	5	(15.63)
None*	13	(14.04)	8	(25.00)

* No other virus was detected in this test.
